# Nanosecond, Nanometer Movies of MEMS/NEMS Dynamics in a Standard SEM Using Time‐Tagged Secondary Electrons

**DOI:** 10.1002/smtd.71029

**Published:** 2026-09-09

**Authors:** Joel Rehmann, Constantin Schalast, Karsten Kunze, Jan Rhensius, Yves Acremann

**Affiliations:** ^1^ Laboratory for Solid State Physics ETH Zürich Zürich Switzerland; ^2^ ScopeM ETH Zürich Zürich Switzerland; ^3^ QZabre AG Zürich Switzerland

**Keywords:** MEMS, micro‐ and nanomechanical resonators, nanosecond dynamics, NEMS, phase‐resolved imaging, secondary‐electron time tagging, time‐resolved scanning electron microscopy

## Abstract

Dynamic phenomena at the micro‐ and nanoscale underpin technologies ranging from micro‐ and nanoelectromechanical systems (MEMS/NEMS) actuators and sensors to quantum instrumentation and communication hardware. In this paper, we demonstrate that time‐resolved full‐field scanning electron microscopy (SEM) for periodically driven micro‐ and nanostructures offers access to their spatiotemporal dynamics. This is achieved using a conventional SEM operated with its continuous electron beam and combined with a multi‐channel plate (MCP) with a time‐to‐digital converter (TDC) for time‐resolved measurements. Effective frame rates from $3.2\times 10^{6}$ to $1.2\times 10^{9}$ frames per second are reached and motion tracking with ≈3nm spatial and ≈3ns temporal jitter is demonstrated, enabling access to both micrometer‐scale and nanometer‐scale motion. By bringing high temporal resolution to widely available electron microscopes without invasive column modifications, this approach enables a broad range of experiments on dynamic micro‐ and nanomechanical systems.

## Graphical abstract

For the observation of micromechanical devices, a fast imaging technique with high temporal resolution is needed. Here, we demonstrate that a standard scanning electron microscope can be converted into a time‐resolving instrument using a fast detector and a time‐to‐digital converter. We evaluate its performance on a shear‐mode crystal oscillator as well as an AFM cantilever.

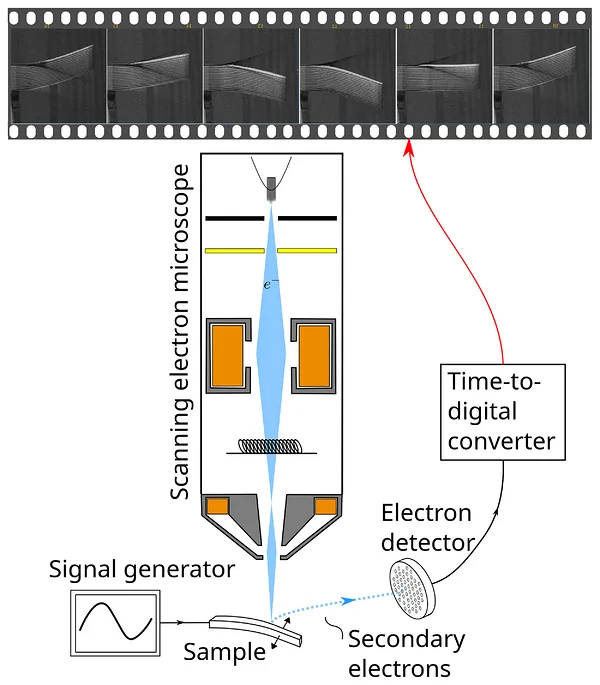

## 1 Introduction

Dynamic mechanical micro‐ and nanosystems are central to modern technology, spanning MEMS/NEMS sensors [1, 2, 3, 4, 5], actuators [6, 7], and emerging platforms such as optomechanics and quantum technologies [8], advanced optical imaging [9], mesoscopic physics [10], scanning force microscopy [11], graphene‐based MEMS [12, 13], and carbon‐nanotube nanomotors [14]. Understanding and optimizing these devices requires in‐situ access to their full spatiotemporal dynamics rather than static snapshots.

The SEM is one of the most widely available tools for imaging micro‐ and nanostructures with high spatial resolution. Its primary imaging modality relies on secondary electrons (SEs), whose yield is sensitive to surface topography and local work functions [15]. Extending SEM imaging from static micrographs to movies enables direct insight into device operation and failure modes [16, 17, 18, 19]. Time‐resolved SEM imaging can supplement or replace optical methods such as Doppler vibrometry [20, 21] and various interferometric methods [22, 23, 24, 25, 26, 27], especially for imaging in‐plane movements, which are difficult to observe with interferometric methods [28].

Established approaches of time‐resolved SEM predominantly rely on stroboscopic (pump–probe) schemes that require pulsing the primary electron beam and synchronizing it to the sample excitation [29, 30, 31, 32]. These methods can demand substantial modifications of the electron column (e.g., fast beam blankers or pulsed‐laser photocathodes) and may compromise routine operation through reduced current, stability constraints, or added complexity [33, 34, 35]. A broadly usable alternative is therefore needed. In particular, a minimally invasive method is desirable that retains continuous‐beam SEM operation while providing nanosecond‐scale access to sample dynamics.

A continuous‐beam paradigm addresses this need by time‐tagging detected electrons and correlating their arrival times with the phase of a periodic excitation. The only modification to the electron microscope is the addition of a time‐resolved detector (plus external timing electronics). We previously demonstrated this concept in a transmission electron microscope (TEM) using a fast detector to resolve the magnetic‐vortex gyration with 122 ps temporal resolution [36]. Here, we transfer this methodology to SEM by supplementing a standard Everhart–Thornley detector with a fast microchannel plate (MCP) detector for time‐resolved SE detection. The MCP, together with a time‐to‐digital converter (TDC), serves as a single‐pixel time‐tagging detector. Spatial resolution is provided by an external scan generator, which enables time‐resolved maps of the SE signal. The sample is excited by a periodic electric signal, and the TDC data are mapped to the excitation phase. This procedure yields a movie of the process under observation. This technique is similar to a pump‐probe experiment as the actual imaging process is much slower than the observed dynamics. In addition, the sample needs to react deterministically to the excitation signal: fluctuating systems cannot be observed. This contrasts to full‐field imaging techniques using cameras or to single‐shot imaging that use pulsed x‐ray sources, for example [37, 38]. The frame rate of up to $1.2\cdot 10^{9}$ frames per second stated in this paper is therefore an *effective frame rate*, which is not related to the actual scan speed of the microscope.

We validated the method on two mechanical resonators: an AFM cantilever mounted on a quartz tuning fork (driven in a flexural mode) and a quartz reference clock oscillator operated in shear mode. Together, these benchmarks demonstrate (i) time‐resolved SEM movies of micromechanical and nanomechanical motion using a continuous primary beam, (ii) compatibility with standard SEM imaging conditions without invasive column modifications, and (iii) quantitative access to mode shapes and phase evolution across a device.

## 2 Results and Discussion

### 2.1 Performance Characterization

We monitor the trajectories of gold nanoparticles with a nominal diameter of 25 nm deposited on a shear‐mode quartz oscillator (Figure 1) to quantitatively assess the temporal and spatial resolution limits of the time‐tagging methodology in the experimental configuration proposed here. Under these conditions, we demonstrate low nanosecond temporal jitter and low nanometer spatial jitter. A symbolic representation of the shear mode quartz oscillator can be found in Figure 1A. A sinusoidal potential difference between a contact on the top surface of the quartz and a contact on the bottom surface leads to shear mode oscillation. For this measurement, the quartz oscillator with its fundamental mode at 12 MHz was electrically excited by a function generator in a spurious mode at a frequency of 12.588 MHz as it shows an elliptic movement, ideal for demonstration purposes (Figure 1G). A movie with 96 frames in one period was recorded, resulting in a frame rate of $1.2\cdot 10^{9}$ frames per second. Then, a quadratic polynomial was fitted to the position of a gold nanoparticle to find its center as a function of time. In Figure 1B the measured positions of the tracked gold nanoparticle as a function of time are displayed and fitted with two sine functions. The tracked gold nanoparticle and the quartz crystal at that location attain a maximum velocity of 2.7 m/s and are subjected to a maximum acceleration of $2.2\cdot 10^{8}$ m/s^2^. The distribution of the differences between the measured positions and the fitted sine functions serves as an estimator of the temporal and spatial jitter of the measurement configuration. Assuming a normal distribution, we measure a temporal jitter of $\sigma_{t}\approx 3$ ns and a spatial jitter of $\sigma_{s}\approx 3$ nm (Figures 1C,D). We assume that most of this measured jitter originates from the counting noise on the individual pixels due to a low electron count. The electron count per pixel was limited by a finite electronics acquisition rate and a spatial drift of the microscope of ≈ 5 nm/min, which limited the acquisition time. Due to the nature of the time‐tagging detection scheme, which records all phases within a single oscillation period simultaneously, this drift blurs the image in the drift direction but should not distort the observed motion. The native temporal resolution is mainly determined by the flight time spread of the electrons from the sample to the detector: the timing jitter between the function generator output driving the sample and the reference signal is 250 ps. The time resolution of the TDC accounts for another 82.3 ps. The MCP detector adds <85ps time jitter. In contrast, the flight time spread for the direct path from the sample to the detector (assuming an energy spread of 5 eV) is 600 ps. This spread increases if we include different emission angles.

**Figure 1:**
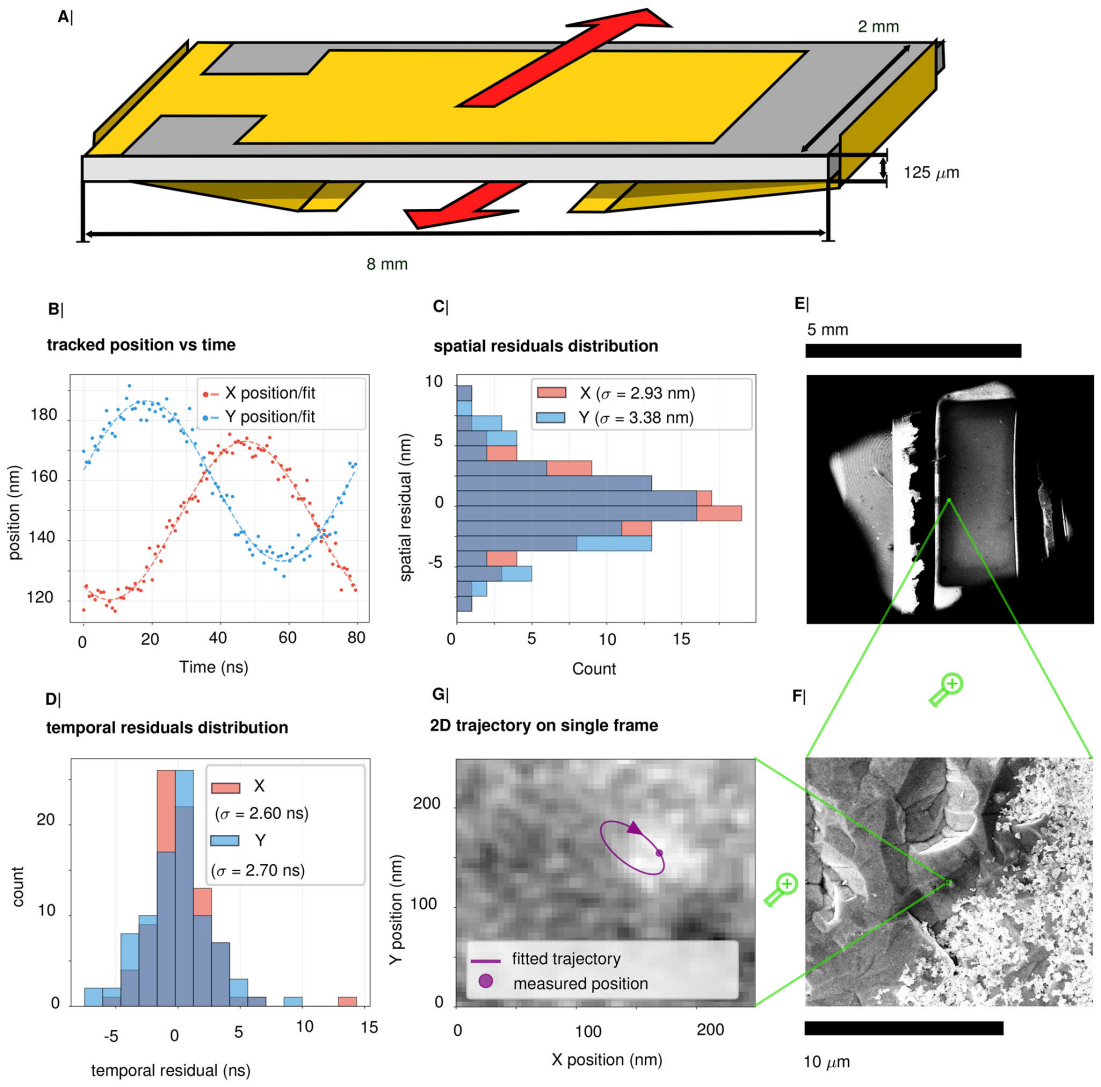
Benchmark of temporal and spatial precision on a driven quartz oscillator. Figure (A) shows our demonstration sample and illustrates oscillation in shear mode. Figure (B) shows the measured motion of a point on the top contact of the shear‐mode quartz oscillator in the x and y axes, together with sine fits. Figure (C) shows a histogram of the spatial residuals (difference between the fit and the data), and figure (D) shows the corresponding residuals in time. Figures (E) and (F) show the position of the tracked point. Figure (G) shows a single frame (50 × 50 pixels with 5 nm pixel size) from the recorded movie with the tracked trajectory overlaid.

To rule out any influence of time‐dependent electric fields on the trajectory of the primary electrons, the examined top contact was held at ground potential while the bottom contact was electrically driven. In addition, other eigenmodes of the shear‐mode quartz oscillator were detected at distinct resonance frequencies, exhibiting displacements along different directions and with different polarisations (for example, see the spurious mode at 12.612 MHz in the supplementary material). No detectable movements were observed for out of resonance excitation. These observations substantiate the fact that the recorded displacements are indeed mechanical in origin and not deflections of the primary electron beam induced by electric fields.

### 2.2 Application Example: Time‐Resolved Imaging of an Atomic Force Microscopy (AFM) Cantilever

In the following, we demonstrate the performance of the instrument by imaging an AFM cantilever attached to a quartz tuning fork. Recorded movies (see supplementary material) of the cantilever demonstrate that our time‐tagging scheme enables quantitative characterization of the 2D in‐plane dynamics of a MEMS device from a single measurement, thereby facilitating precise modeling of the physical system. The movies were reconstructed with 100 frames per oscillation period for all modes. Six frames are shown in Figure 2. The SEM was operated at 10 keV beam energy and at a working distance of 23 mm. This working distance was chosen to allow for a larger scan range while avoiding edge artifacts due to electron optical lens abarrations. For the slowest mode, this corresponds to an effective frame rate of $3.22\cdot 10^{6}$ frames per second and an effective time resolution of 311 ns. For the fastest mode, we have an effective frame rate of $5.22\cdot 10^{7}$ frames per second and an effective time resolution of 19.2 ns.

**Figure 2:**
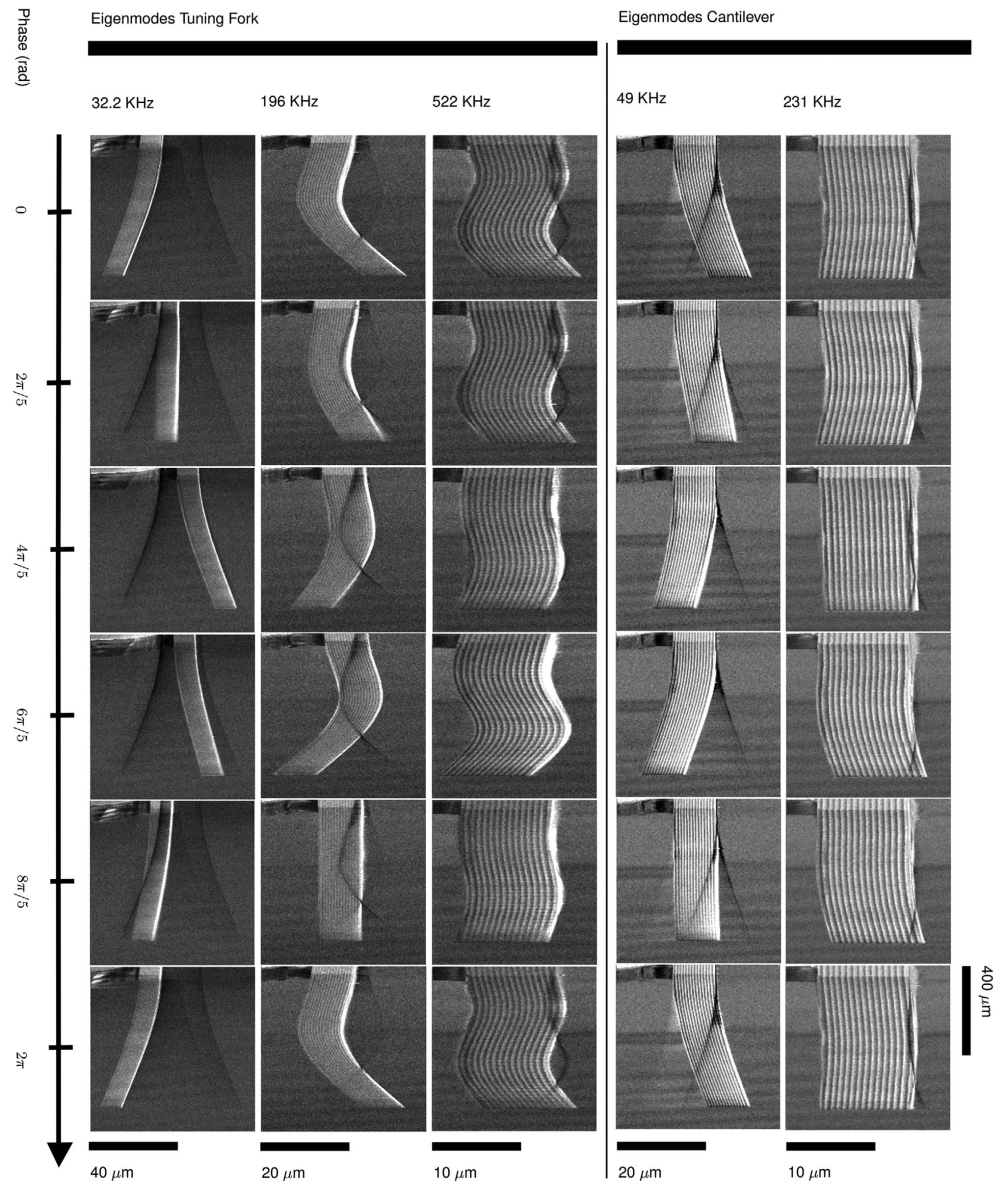
Extracted frames of the recorded movies of the AFM cantilever. The full movies consist of 100 frames per period and are represented here by six frames at a fixed phase in relation to the excitation. Note that the scaling of the x‐ and y‐axes was adapted to best showcase the deflections. The full movie can be found in the supplementary material.

To provide a quantitative representative measure of image quality, we report a contrast‐to‐noise ratio (CNR) for one mode and one phase frame. Taking Poisson‐limited statistics into account, the CNR between the leading edge of the cantilever (used for motion tracking) and the background in the 32.2 kHz mode at phase π/4 is CNR $=\left(\mu_{e}-\mu_{b}\right)/\sqrt{\sigma_{e}^{2}+\sigma_{b}^{2}}\approx 10$. $\mu_{e}=130$ is a conservative lower bound for the counts per pixel on the leading edge, and $\mu_{b}=27$ is the mean counts of the background. The background is mainly caused by secondary electrons from the sample stage. The CNR is phase‐dependent due to the variation in the velocity of the cantilever. The dark counts from the detector are negligible (<20 counts/s) in all the data presented. This corresponds to fewer than 0.05 dark counts in a pixel of a single frame when measuring with a dwell time of 250 ms per position and dividing the data into 100 frames. The brightest pixel in the 32.2 kHz movie recorded an average detection rate of $1.14\times 10^{5}$ electrons/s (integrated over all frames). The electron detection chain can maintain a detection rate of $\approx 5\times 10^{5}$ electrons/s. The bottleneck is the acquisition of the data generated by the TDC, which measures the arrival time of the electrons. Instantaneous peak rates can saturate the data acquisition system. This saturation appears as black lines in Figure 2 and can be mitigated with a reduced electron flux at the cost of longer acquisition times or by implementing improved electronics.

Figures 3A,C show the AFM cantilever mounted on a quartz tuning fork at a $35^{\circ }$ angle. To obtain a sharp image across the full field‐of‐view, the sample stage was tilted by $35^{\circ }$. In Figure 2, we observe the top surface of the cantilever. Notice that the x‐axes of the images are scaled differently compared to the y‐axis to enhance the visibility of the dynamics. We resolve five modes of the combined system: at 32.2, 196, and 522 kHz, we observe the first three modes of the quartz tuning fork to which the AFM cantilever is mounted. At 49 and 231 kHz, we find the eigenmodes of the AFM cantilever itself. A schematic representation of the mode shapes is shown in Figure 3B. We validated the full mode shapes by recording movies of both the tuning fork and the cantilever. The first two eigenfrequencies of the cantilever are located close to the eigenfrequencies of the tuning fork. Hence, the AFM cantilever assumes a similar shape of deflection during forced oscillation in the first two eigenmodes of the tuning fork as it does when the first two eigenmodes of the cantilever are excited. Figure 3D depicts the S‐parameter S21(f) of the device measured with a network analyzer. The eigenmodes of the tuning fork are visible in those purely electric measurements, but due to the small mass of the cantilever, its influence on the electric response of the system cannot be resolved with the network analyzer used.

**Figure 3:**
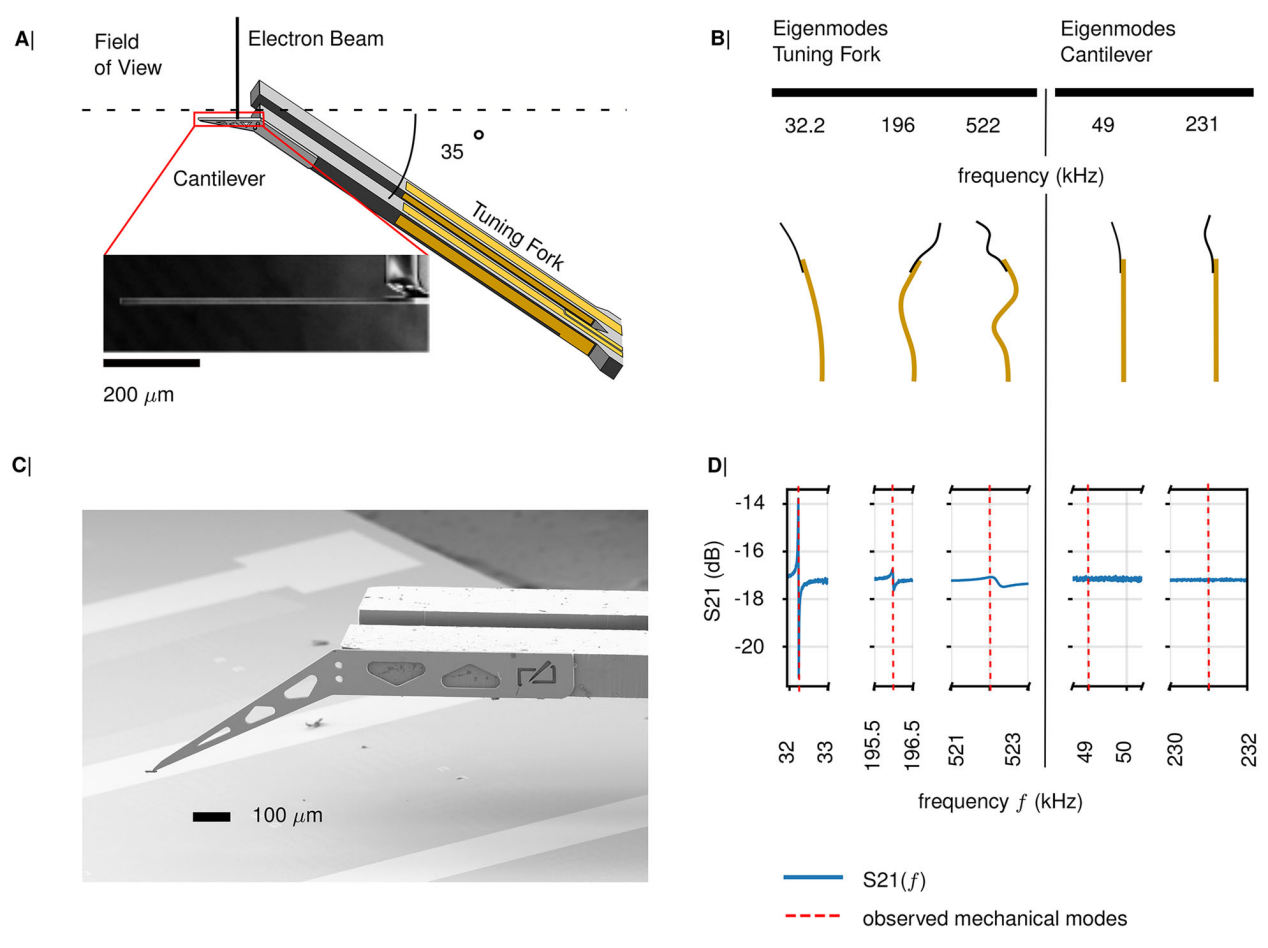
(A) shows the orientation of the AFM cantilever during measurement. The provided SEM image was recorded at 10 keV beam energy with the standard Everhart–Thornley detector. (B) a symbolic depiction of the modes observed in Figure 2. (C) Static scanning electron microscopy (SEM) images of the observed cantilever attached to a piezo tuning fork with a mounted NV center recorded at 5 keV primary electron beam energy on a Zeiss Ultra 55. Image used with courtesy of QZabre. (D) Frequency‐dependent forward transmission $S_{21}\left(f\right)$ of the cantilever–tuning‐fork assembly, measured with a network analyzer. The eigenmodes of the tuning fork are detectable and confirm the mechanical resonances, but those of the cantilever are not detectable.

#### 2.2.1 Physical Modeling

We modeled the in‐plane bending of the AFM cantilever using an Euler‐Bernoulli beam [39, 40]. The presence of cutouts on the cantilever induces a spatially varying effective thickness of the beam, denoted by h(x), which in turn gives rise to a position‐dependent area moment of inertia I(x) and a spatially varying mass per unit length μ(x). The beam width is constant at w=10μm. Figure 4A presents a side view of the cantilever recorded with an SEM including the cutouts, while Figure 4B depicts the area moment of inertia obtained directly from this geometry. The cantilever model is driven by the base motion of the tuning‐fork, which was extracted from the cantilever movies in Figure 2. We solve the resulting boundary‐value problem to obtain the deflection profile $u\left(x,t\right)=\hat{u}\left(x\right)e^{i\omega t}$ at the drive frequency ω. Comparison of the simulated deflection to the experimentally extracted shapes (Figure 4C) shows that a single calibration parameter held constant for all modes is sufficient to describe the system. This parameter is an effective Young's modulus $E_{\mathrm{eff}}$ = 65 GPa, which accounts for residual uncertainties (e.g., geometry, clamp compliance, and neglected torsion). Notice that this constant is an effective value that does not match the material constant. To quantify the quality of our model, we calculate the coefficients of determination $R^{2}$ for the different modes. We obtain the coefficients of determination of $R_{32.2\;\mathrm{kHz}}^{2}=0.999$, $R_{196\;\mathrm{kHz}}^{2}=0.996$, and $R_{522\;\mathrm{kHz}}^{2}=0.950$ when comparing the model predictions with the cantilever mode shapes measured on the right edge of the cantilever. This agreement demonstrates that the time‐resolved SEM movies faithfully capture the true mechanical dynamics and can be used for quantitative mode‐shape analysis. The complete derivation, boundary conditions, and parameter definitions are given in the supplementary material. In general, these results show that time‐tagged SEM imaging provides quantitative access to in‐plane motion, complementary to optical stroboscopic interferometry for out‐of‐plane dynamics, as demonstrated in [24].

**Figure 4:**
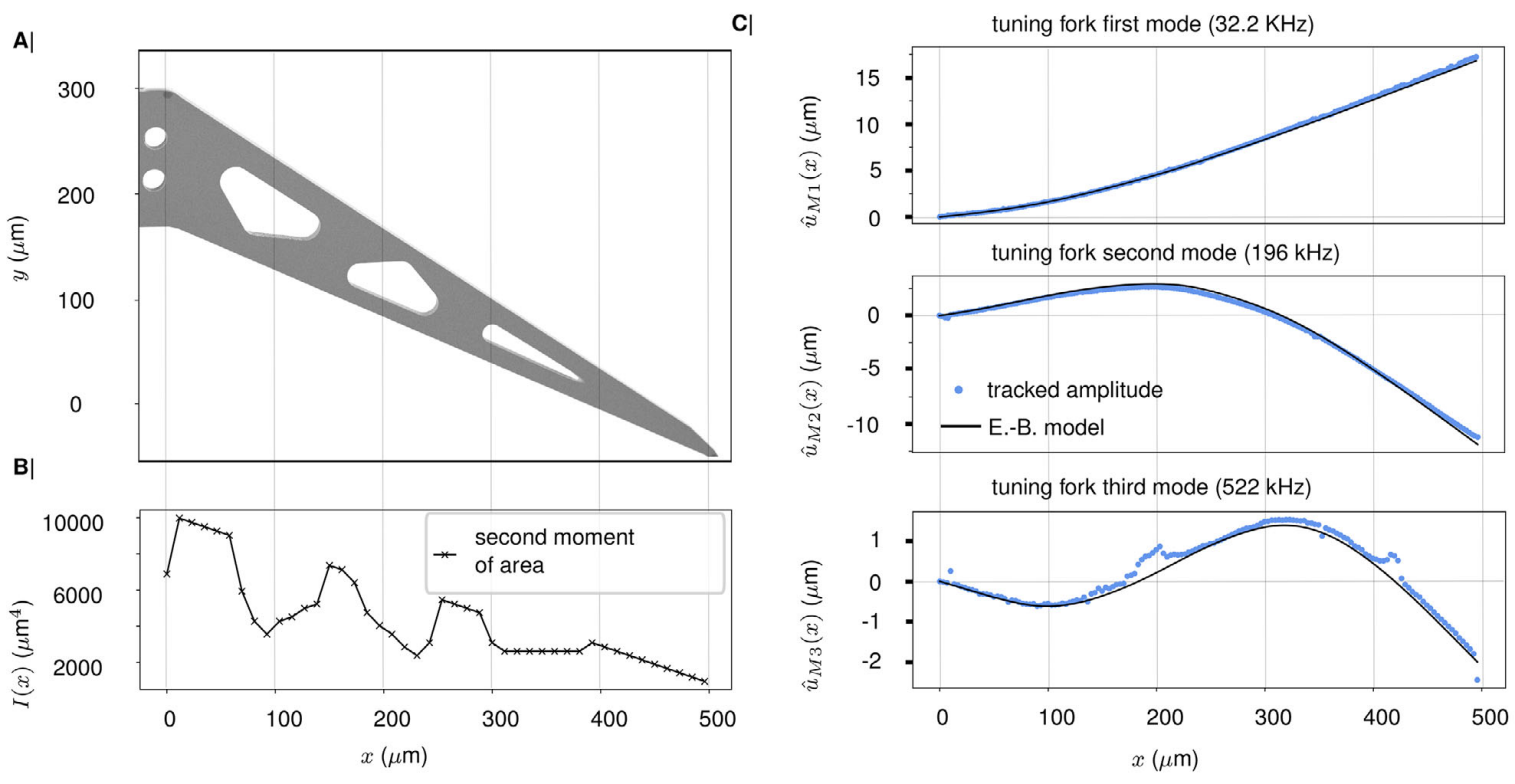
(A) shows an SEM image cut out of the AFM cantilever to illustrate the origin of the second moment of area I(x) and the reference frame used in the numeric Euler‐Bernoulli model. (B) depicts the second moment of area I(x) derived from A. (C) Comparison of the x‐dependent amplitude as reconstructed using motion tracking from Figur 2 and as simulated with a numeric Euler‐Bernoulli model with I(x) from B and a density distribution μ(x)∝I(x). Notice, that a single effective Young's modulus $E_{\mathrm{eff}}$ = 65 GPa is used for the fit. Its value takes unknown contributions like softening by torsional motion into account.

## 3 Conclusion

We demonstrated continuous‐beam time‐resolved SEM based on time‐tagging of individual secondary electrons and phase‐correlated reconstruction of periodically driven motion. By combining standard raster scanning with a fast MCP detector and timing electronics, this approach turns a conventional SEM into a phase‐resolved movie camera for micro‐ and nanomechanical devices, reaching effective time resolutions down to the nanosecond regime while retaining the continuous‐beam operating mode.

Compared to established stroboscopic time‐resolved approaches in electron microscopy that rely on pulsing the primary beam and performing pump probe experiments [29, 30, 31, 33, 34, 35], our method shifts the timing requirement from beam generation to detection. This removes the need for invasive column modifications and avoids the reduced duty cycle inherent to pulsed‐beam operation. Compared to optical vibrometry and interferometric schemes, SEM‐based contrast is inherently sensitive to lateral changes in edge position and surface features, enabling direct access to in‐plane motion without stringent optical access, alignment, or sample‐specific reflectivity requirements [22, 23, 41].

As we take advantage of the original electron optics of the microscope, its spatial resolution (in our case <3 nm) is maintained. The temporal resolution is 3 ns and we can observe slower processes using a delay range of milliseconds (given by the range of the TDC). In comparison, if the electron beam of an SEM is pulsed, sub‐picosecond time resolution can be achieved [42]. However, this technique is limited in time scan range due to the length of the optical delay line, and the electron flux is lower compared to that of the continuous beam SEM. The situation is similar for ultrafast transmission electron microscopes, except that these instruments provide atomic resolution [16]. However, the transmission geometry leads to additional challenges for in‐operando experiments. For optical methods with nanometer spatial resolution, we can compare our technique with time‐resolved x‐ray microscopy at synchrotron sources. These instruments provide a spatial resolution of up to 20 nm [43]. The temporal resolution is given by the pulse length of the x‐ray pulses of approx. 50 ps. These instruments have been used to study devices in operando [44], but access is limited due to the sparse availability of beam time. By coherent x‐ray diffraction imaging at free electron laser (FEL) sources, it is possible to achieve nanometer resolution. The temporal resolution is limited by the pulse length of the FEL as well as the synchronization with the sample excitation in the low femtosecond range. The high pulse energy is sufficient for single shot imaging [37, 38]. However, access to an FEL is even more limited than access to a synchrotron.

The present implementation has several practical constraints that define its current operating window. First, the reconstruction assumes reproducible, periodically excited dynamics; non‐repetitive transients are not captured in a single acquisition. Second, the achievable acquisition speed is limited by the detected count rate and data‐throughput of the timing chain, which can lead to saturation artifacts at high instantaneous rates, as observed in the cantilever movies. Third, at the highest magnifications and smallest pixel sizes, long dwell times make the measurement increasingly sensitive to instabilities of the microscope itself. The jitter of the function generator may also limit the time resolution. Finally, the timing fidelity is influenced by the secondary‐electron energy spread, the emission angle spread, and the corresponding flight‐time distribution, which may become relevant below the nanosecond regime.

Despite these limitations, the demonstrated capability to extract mode shapes and spatially resolved phase in operando provides a quantitative tool for finding and validating device models. Looking ahead, a higher‐throughput time‐to‐digital converter and detector geometries with improved collection efficiency will immediately translate into shorter acquisition times and higher dynamic range. The concept could be further extended to in‐lens secondary electron detection for time‐resolved voltage‐contrast measurements [33] and to backscattered electron detection, where the suppression of slow secondary electrons can reduce the dispersion of flight‐time and extend the accessible time scale to faster periodic processes.

By enabling nanosecond‐resolved operando SEM movies of in‐plane motion on standard instruments, this method directly supports the development and validation of MEMS/NEMS sensors and actuators, optomechanics, scanning‐probe microscopy platforms, and emerging nanomechanical systems such as graphene‐based MEMS and carbon‐nanotube nanomotors by revealing full‐field mode shapes, phase evolution, and dynamic behavior that static SEM or optical interferometry often cannot access.

## 4 Experimental Section

### 4.1 Electron Detector

Time‐resolved secondary electron detection was performed using a Surface Concept MCPD18 microchannel plate detector mounted on the port conventionally used for an energy‐dispersive X‐ray spectrometer (Figure 5A). The detector consists of a double‐stack MCP with an active diameter of 18 mm and a pore diameter of 10.3 μm as specified by the manufacturer. The MCP is positioned at a distance of 34 mm from the sample and oriented at an elevation angle of 35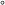. Individual electron events generate voltage pulses of approximately −4 mV amplitude and 2 ns full width at half maximum (FWHM) when measured across a 50 Ω load.

**Figure 5:**
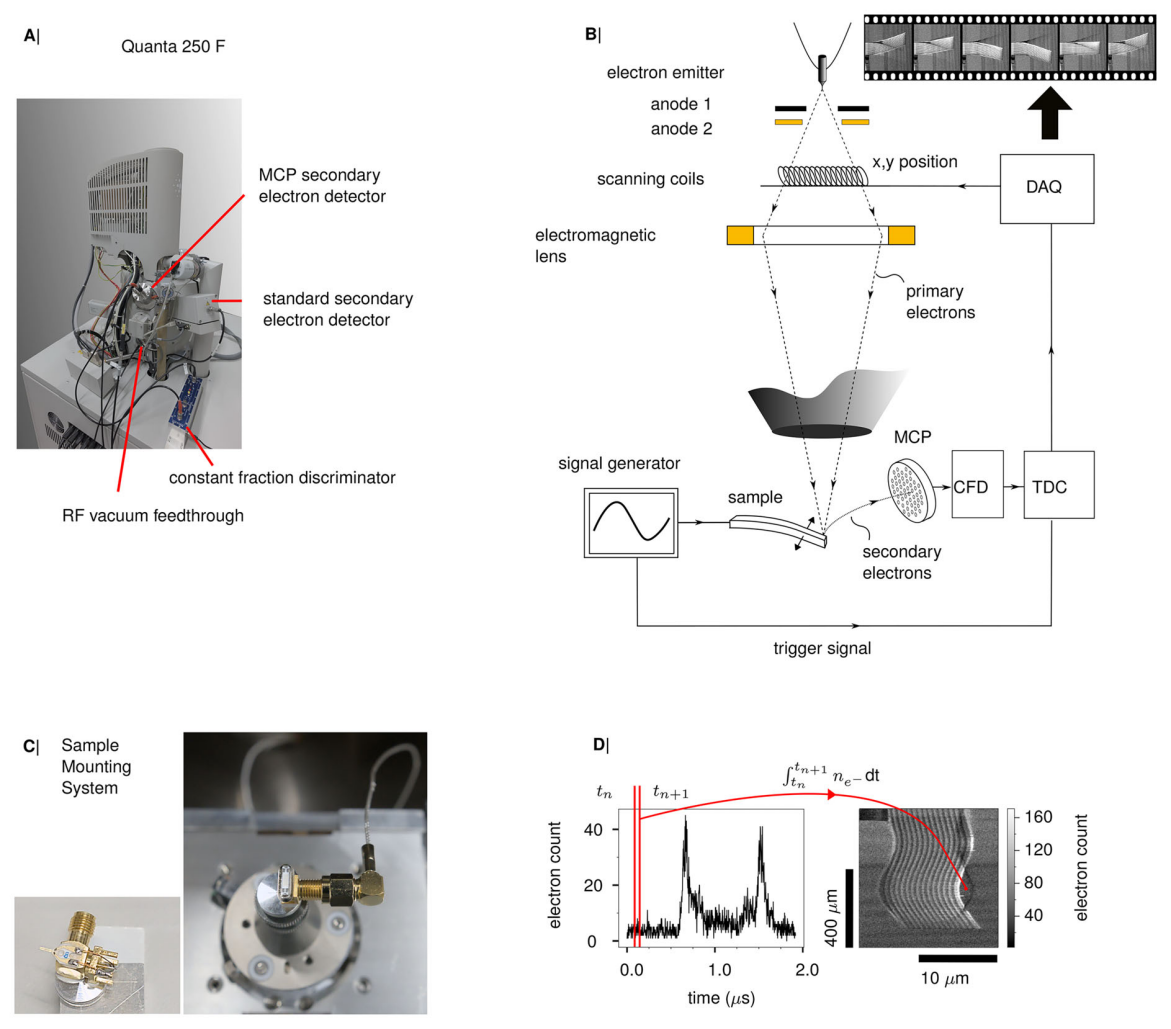
(A) Overview of the Quanta 250 F equipped with the time‐tagging MCP serving as a secondary electron detector. (B) Symbolic overview over the data acquisition system (DAQ). (C) Both the AFM cantilever and the quartz reference oscillator are connected via SMA RF coaxial cables to a signal generator. (D) Illustration of the time‐binning and phase‐resolved reconstruction procedure. Electron arrival times are recorded with 82.3 ps resolution and subsequently grouped into reduced time bins for efficient storage. For periodic excitations, the binned data of each pixel are redistributed into phase frames to form a time‐resolved image sequence.

A voltage of 1850 V is applied throughout the MCP stack, providing sensitivity to single electrons and a low dark‐count rate of <20 electrons/second. In addition, a voltage of 200 V is applied to the surface to collect secondary electrons from the sample. The optimal front‐surface potentials depend on the geometry of the sample.

A conventional Everhart–Thornley (ET) detector was operated in parallel to the MCP. Simultaneous operation reduces the MCP count rate by 0–20%, depending on the voltage of the ET grid (−150V to 250 V), the MCP front potential and the working distance (10 to 25 mm). The ET detector was used primarily for rapid visual alignment, focusing, and navigation due to its higher imaging efficiency, while the MCP provided time‐resolved single‐electron detection.

### 4.2 Timing Electronics

An overview of the data acquisition system can be found in Figure 5B. Analog pulses from the MCP were processed by a constant‐fraction discriminator (CFD) that generated LVTTL‐level timing pulses with minimized time walk. Arrival times were recorded relative to a trigger signal (5V CMOS level, rise time: 3.5 ns) from a function generator (Hameg HMF 2550) driving the sample excitation. The time base of the function generator is stable within 1 ppm according to the manufacturer. Time digitization was performed using a Surface Concept SC‐TDC 1000 S‐Series time‐to‐digital converter (TDC), providing a bin width of 82.3 ps and an effectively unlimited measurement range. The TDC bin size can be converted into an effective sampling rate of 12.2 GHz. Due to the Nyquist sampling theorem, the quantization noise of the TDC can therefore be neglected for our application. The typical time resolution of our detector is specified as < 85 ps by the manufacturer [45]. An additional coaxial SMA radio‐frequency vacuum feed‐through allowed direct electrical excitation of the sample (Figure 5C).

### 4.3 Beam Scanning and Time‐Resolved Data Acquisition

The beam was scanned across the sample by controlling an external scanning unit (DISS 6 by Point Electronics) using software. For each pixel, every MCP‐detected electron was individually time‐tagged, providing both its spatial origin on the sample and its detection time. To quantify the acquisition time required to obtain the presented data set, we consider two representative examples. For the movie corresponding to the 12.588 MHz mode of the shear‐mode quartz oscillator, an integration time of 150 ms per pixel was used. Including an additional overhead of 20 ms per pixel, this yielded a total acquisition time of approximately 7 minutes for the targeted 50×50‐pixel movie.

For the movie corresponding to the 522 kHz mode of the AFM cantilever, an integration time of 250 ms per pixel was used. Taking into account an overhead of 20 ms per pixel, the total acquisition time for 200×200 pixels amounted to approximately 3 hours. This overhead is dominated by the time to starting a new acquisition with the TDC. In addition, the readout of the histogram from the TDC as well as the communication to set the new electron beam position adds to the dead time. A future fully integrated scanning and TDC system will be able to operate at a higher scan rate.

### 4.4 Time‐Binning Strategy and Reconstruction of Periodic Motion

A visual explanation for the creation of movies via time‐binning is given in Figure 5D. We record pixel by pixel. All the secondary electrons reaching the MCP while observing this location are time‐tagged. After recording these arrival times for multiple excitation periods to gather sufficient statistics, we obtain a histogram as shown in Figure 5D of the TDC with a time binning corresponding to the resolution of the TDC of 82.3 ps. To observe our mechanical systems, we want a coarser time binning. We choose a frame rate $f=\frac{1}{\Delta t}$ that we want to achieve, while all electrons that arrive within the period $\Delta t=t_{n+1}-t_{n}$ will then form one pixel in one frame of the final movie. The histogram in Figure 5D shows the bright edge of the cantilever crossing one point in the image on the right. On the right, a frame is shown that was extracted from the time span $\left[t_{n},t_{n+1}\right]$ marked on the histogram. This procedure produced a phase‐resolved sequence of secondary‐electron images that captured the periodic motion of the sample.

We illustrate this process using the example of the third flexural mode of our AFM cantilever at 522.25 kHz; this corresponds to 23266 TDC bins per pixel for one full oscillation period. If our final movie should consist of 100 frames per oscillation period ($5.22\cdot 10^{7}$ frames per second), we measure the intensity at one pixel in one frame by counting the electrons arriving within $\Delta t=t_{n+1}-t_{n}$ = 19.1 ns. Thus, 233 TDC bins are mapped to one pixel in one frame.

## Supporting information


Supporting Information

Supporting Information

## Data Availability

Supplementary notes are provided. Furthermore, raw data and analysis code can be found in https://doi.org/10.3929/ethz‐c‐000799039.
