# Supplementary material for: Nanosecond, Nanometer Movies of MEMS/NEMS Dynamics in a Standard SEM Using Time‐Tagged Secondary Electrons
Source: Small Methods. 2026 Sep 9;10(19):e71029. doi: 10.1002/smtd.71029 (PMC13646037; doi:10.1002/smtd.71029)
Supplement: Supplementary file 1 [file SMTD-10-e71029-s002.pdf]

Supplementary Material for: Nanosecond, nanometer movies of MEMS/NEMS dynamics in a standard SEM using time-tagged secondary electrons

Supplementary Notes 1: Tracking of the cantilever mode

For extraction of the mode shape, we applied a threshold to the data, turning them into binary images. In there, we searched for the position of the right-hand edge. Before that, isolated pixels of value 1 (caused by noise) were removed.
To estimate the tracking error, we subtract the extracted position of the cantilever u from neighboring scan lines, as these are independent measurements

$$\Delta u_n = u_n - u_{n-1}. \tag{1}$$

Here, n denotes the scan line. The resulting Δu_n is the tracking error plus the derivative of the shape of the mode $\mathrm{d}\hat{u}/\mathrm{d}x$. To remove the derivative and obtain the error, we subtract the low-pass filtered difference $< \Delta u_n >$:

$$e_n = \Delta u_n - < \Delta u_n > . \tag{2}$$

This procedure results in a standard deviation of the tracking of 52 nm (fork mode 1), 37 nm (fork mode 2), 89 nm (fork mode 3), 47 nm (cantilever mode 1) and 28 nm (cantilever mode 2).

Supplementary Notes 2: Modelling of the AFM cantilever

This supplementary note provides the full derivation of the simplified cantilever model demonstrated in the main text.
We assume that the Euler–Bernoulli beam equation describes the cantilever dynamics [1, 2]:

$$\frac{\partial^2}{\partial x^2}\left(EI(x)\frac{\partial^2 u}{\partial x^2}\right) = -\mu(x)\frac{\partial^2 u}{\partial t^2} + f(x,t). \tag{3}$$

with the Young's modulus E, the second moment of inertia I and the mass per unit length μ. u is the deflection of the beam and x the coordinate along the beam. f is the external force density applied perpendicular to the beam. This model neglects the twisting motions of the cantilever that can arise from the slanted mounting geometry.
To incorporate the excitation (a nearly sinusoidal translation and rotation of the clamped end), we analyze the system in the accelerated reference frame of the excited end. In that frame, the excitation enters as a fictitious force density $f(x,t) = -\mu(x)\omega^2(A_0 + x\cdot\alpha_0)e^{i\omega t}$, which gives the following:

$$\frac{\partial^2}{\partial x^2}\left(EI(x)\frac{\partial^2 u}{\partial x^2}\right) = -\mu(x)\frac{\partial^2 u}{\partial t^2} - \mu(x)\omega^2(A_0 + x\cdot\alpha_0)e^{i\omega t}. \tag{4}$$

Assuming a linear response at the drive frequency, we use the Ansatz

$$u(x,t) = \hat{u}(x)e^{i\omega t}. \tag{5}$$

Inserting Eq. (5) into Eq. (4) gives the time-independent boundary value problem

$$\frac{\partial^2}{\partial x^2}\left(EI(x)\frac{\partial^2 \hat{u}}{\partial x^2}\right) = \mu(x)\omega^2\big(\hat{u}(x) - A_0 - x\cdot\alpha_0\big). \tag{6}$$

We apply the standard clamped–free boundary conditions. At the clamped end ($x = 0$) the deflection and slope vanish in the accelerated frame,

$$\hat{u}(0) = 0, \tag{7}$$

$$\frac{\partial}{\partial x}\hat{u}(0) = 0, \tag{8}$$

while at the free end ($x = L$) the bending moment and shear force vanish,

$$\frac{\partial^2}{\partial x^2}\hat{u}(L) = 0, \tag{9}$$

$$\frac{\partial^3}{\partial x^3}\hat{u}(L) = 0. \tag{10}$$

The area moment of inertia $I(x)$ is computed from the cantilever geometry (constant width $w = 10$ μm and an x-dependent effective height $h(x)$), $I(x) = \frac{w\,h(x)^3}{12}$. The mass per length is approximated as $\mu(x) = w\,h(x)\,\rho_{\mathrm{Si}}$ with $\rho_{\mathrm{Si}} = 2330$ kg/m^3 [3]. Solving Eq. (6) numerically yields $\hat{u}(x)$. Comparing the model to the experimentally extracted deflection profiles for off-resonant driven oscillations shows a systematic amplitude mismatch if we use the tabulated Young's modulus for $\langle 100 \rangle$ silicon, $E = 130$ GPa [4]. We therefore introduce an effective Young's modulus E_{eff} as a single calibration parameter; best agreement for the three driven responses shown in the main text is obtained for $E_{\mathrm{eff}} = 65$ GPa. Importantly, E_{eff} should not be interpreted as a change of the intrinsic elastic modulus of silicon; rather, it absorbs model and parameter uncertainties (e.g., uncertainty in $h(x)$, deviations from Euler–Bernoulli assumptions, torsion due to slanted mounting, and clamp compliance/coupling to the tuning fork).

Supplementary Notes 3: Bending moment and shear force of cantilever

Through the Euler-Bernoulli model of the cantilever, we can obtain an estimate for the internal bending moment and the shear force using the equations

$$M = -E \cdot I \cdot \frac{\mathrm{d}^2 u}{\mathrm{d}x^2}$$

$$Q = -\frac{\mathrm{d}}{\mathrm{d}x}\left(E \cdot I(x) \cdot \frac{\mathrm{d}^2 u}{\mathrm{d}x^2}\right).$$

For the first three tuning fork modes, the following bending moments and shear forces are received. The clamping moment and force can be read from the leftmost values.

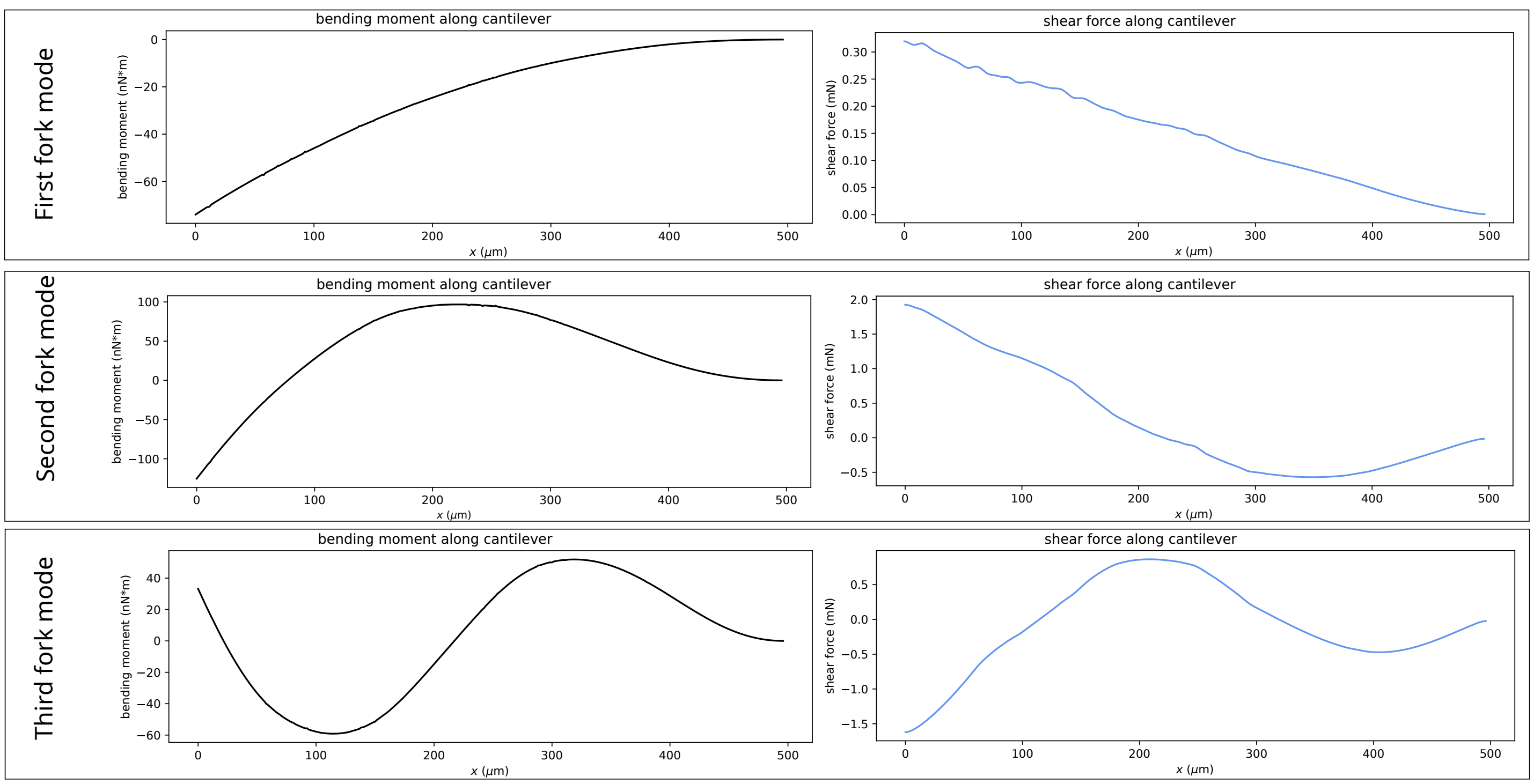

Figure 1: Bending moment and shear forces in the cantilever.

Supplementary Notes 4: Additional Measurement on Shear Mode Quartz Oscillator

We provide an additional measurement of a spurious mode at $f = 12.614$ MHz that does not show elliptic motion and a different overall direction of the oscillation. This indicates that the measured effects in our experiment do not originate from the deflection caused by the electric fields. However, potential deflection effects from electric fields need to be evaluated when using this technique, depending on the sample.

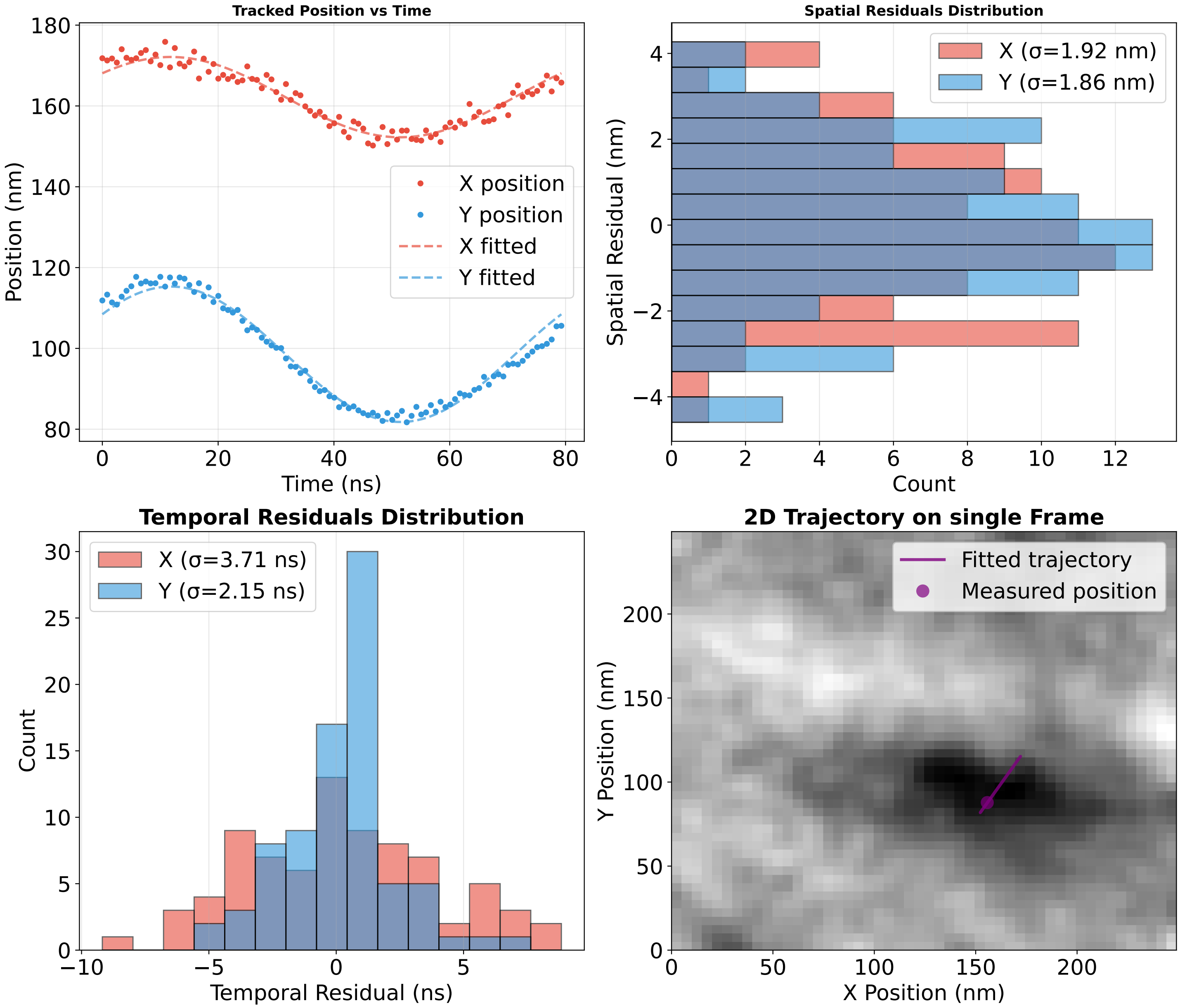

Figure 2: Measurement of a spurious mode at $f = 12.614$ MHz of the shear mode quartz oscillator.

Supplementary Notes 5: Details on Detection of Motion on the Shear Mode Quartz Oscillator

The movies were recorded with a beam energy of 10 keV at a working distance of 10 mm. A frame size of 50x50 pixels with a pixel size of 5 nm by 5 nm was chosen. The datasets were binned to 96 frames. The following fitting parameters were found when fitting sine functions to the two presented modes

Table 1: Fitted parameters for the two modes at f_1 and f_2.

Mode	Component	Amplitude [nm]	Phase [rad]	Offset [nm]
$f_1 = 12.588$ MHz	x	26.34 ± 0.42	-2.17 ± 0.02	146.74 ± 0.30
	y	26.64 ± 0.50	0.14 ± 0.02	159.90 ± 0.40
$f_2 = 12.614$ MHz	x	9.94 ± 0.28	0.64 ± 0.03	162.18 ± 0.20
	y	16.76 ± 0.27	0.63 ± 0.02	98.55 ± 0.20

Supplementary Notes 6: Calibration procedures

The frequency of the function generator used for sample excitation was calibrated by the manufacturer. Its time base is a temperature-compensated crystal oscillator with 1ppm accuracy. We used the length of the cantilever to calibrate the magnification of the SEM. This was cross-checked with the factory calibration of the microscope. The size of the TDC time bin is based on a crystal oscillator.

References

[1] A. N. Cleland, *Foundations of Nanomechanics*, Springer Berlin Heidelberg, **2003**.

[2] S. P. Timoshenko, J. M. Gere, W. Prager, *Journal of Applied Mechanics* **1962**, *29*, 1 220.

[3] ChemBK Database, Silicon monocrystalline, **2024**, URL `https://www.chembk.com/en/chem/Silicon%20Monocrystalline`, Density reported as 2.33 g/cm^3 at 25 °C. Accessed: 2026-02-17.

[4] M. A. Hopcroft, W. D. Nix, T. W. Kenny, *Journal of Microelectromechanical Systems* **2010**, *19*, 2 229.
